# Association between body condition score, testicular haemodynamics and echogenicity, nitric oxide levels, and total antioxidant capacity in rams

**DOI:** 10.1186/s13620-023-00235-y

**Published:** 2023-03-09

**Authors:** Hossam R. El-Sherbiny, Amr S. El-Shalofy, Haney Samir

**Affiliations:** Theriogenology Department, Faculty of Veterinary Medicine, Cairo University, Giza, 12211 Egypt

**Keywords:** Breeding soundness evaluation, Obesity, Rams, Oxidative stress, Testicular blood flow

## Abstract

Higher body fatness adversely affects metabolic and hormonal homeostasis. The present work aimed to evaluate the association between body condition score (BCS) and haemodynamic pattern and echogenic appearence of the testes as well as nitric oxide (NO) levels and total antioxidant capacity (TAC). For that, fifteen Ossimi rams were blocked according to their BCS into a lower BCS group (L-BCS:2–2.5; *n* = 5), medium BCS group (M-BCS:3–3.5; *n* = 5), and higher BCS group (H-BCS:4–4.5; *n* = 5). Rams were examined for testicular haemodynamics (TH; Doppler ultrasonography), testicular echotexture (TE; B-mode image software analysis), and serum levels of NO and TAC (colorimetric). Results are presented as means ± standard error of the mean. There was a significant (*P* < 0.05) difference in the resistive index and pulsatility index means among the groups under experimentation, being the least in the L-BCS group (0.43 ± 0.02 and 0.57 ± 0.04, respectively) compared to the M-BCS (0.53 ± 0.03 and 0.77 ± 0.03, respectively) and H-BCS rams (0.57 ± 0.01 and 0.86 ± 0.03, respectively). Among blood flow velocity measurements [peak systolic, end-diastolic (EDV), and time-average maximum], only EDV showed significant (*P* < 0.05) higher values in the L-BCS group (17.06 ± 1.03 cm/s) compared to M-BCS (12.58 ± 0.67 cm/s) and H-BCS (12.51 ± 0.61 cm/s) groups. Regarding the TE results, there were no significant differences among the examined groups. There were significant differences (*P* < 0.01) in the concentrations of TAC and NO among the groups under experimentation, in which the L-BCS rams had the highest levels of TAC and NO in their sera (0.90 ± 0.05 mM/L and 62.06 ± 2.72 μM/L, respectively) than the M-BCS (0.058 ± 0.05 mM/L and 47.89 ± 1.49 μM/L, respectively) and H-BCS rams (0.45 ± 0.03 mM/L and 49.93 ± 3.63 μM/L, respectively). In conclusion, body condition score is associated with both testicular hemodynamic and the antioxidant capacity in rams.

## Introduction

Animal production faces many major challenges (i.e., climatic, nutritional, and pollution). Improving reproductive efficiency plays a major role in helping with such challenges. The male component of reproductive performance is equally as important as the female in the breeding industry [1]. Breeding soundness examination is a major determinant for recruitment of the males fit for inclusion in the reproductive cycle [2]. Nowadays, intensive production systems are seeking well-defined protocols for the selection of superior-quality males for breeding.

The testis is a highly functioning metabolic organ, in which the production processes (spermatogenesis and steroidogenesis) require a regular supply of nutrients and oxygen via the bloodstream [3]. Therefore, control of blood perfusion to and from the testes is beyond critical for their optimum function [4]. Testicular blood flow is governed by a multitude of factors including age, environment, health status, hormonal balance, and nutrition [5, 6]. A body of evidence has reported a strong relationship between testicular blood flow and testicular competence including steroid production, semen quality, and fertilizing potential [7–10].

Excess body fat alters male physiological homeostasis through hormonal changes, higher scrotal temperature, oxidative stress, and toxin accumulation which perturbates male reproductive performance [1, 11]. Furthermore, obesity impacts male sexual activity through dysregulation of hypothalamic-pituitary-gonadal access [12], a decrease in sex hormone binding, and an alteration of the ratio between testosterone and estrogen [13]. This is likely mediated via the hyper-insulinemia noted in obese animals [14]. In addition, higher leptin concentrations in obese males are inversely correlated with available free testosterone [1]. Moreover, plenty of reports indicate a strong positive relationship between obesity and higher free radicals generation, oxidative and metabolic stress, and lipid peroxidation [15]. Reactive oxygen species (ROS) compulsively bind with nitric oxide (NO), a blood flow modulator, forming a highly pro-oxidant peroxynitrite and consequently diminishing NO bioavailability resulting in lower tissue perfusion [16, 17]. Based on the above-mentioned evidence, it was hypothesized that rams of various body conditions scores would have a differing antioxidant capacity, testicular tissue perfusion, and echotexture. Thus, the hypothesis was tested by evaluation of testicular blood flow (Doppler ultrasonography), testicular echotexture (pixel intensity), and serum concentrations of NO and total antioxidant capacity (TAC) in rams with different body condition scores.

## Materials and methods

### Animals and husbandry

The present study was conducted at the Faculty of Veterinary Medicine, Cairo University, Egypt (30.0276°N, 31.2101°E) in January 2022, following accreditation of the ethical committee of animal handling and care (Protocol no: VetCU 200,092,022,482). Rams were housed in a barn under natural daylight (10.5–11 hours) and air temperature (12–21 °C). They were deemed clinically normal based on clinical examination (i.e., rectal temperature, respiratory and heart rate), reproductive organs (ultrasonography), and cardiovascular system (capillary refilling time). They were fed [Alfalfa blocks (*Medicago sativa*) and concentrates], given free access to water*,* and routinely vaccinated against endemic diseases in Egypt (General Authority of Veterinary Services) including rift-valley fever, foot and mouth disease, sheep pox, and clostridial diseases.

### Experimental design

#### Study type and inclusion criteria

This study was designed as a cohort study for observation of the association between BCS and testicular haemodynamic and echogenic patterns as well as serum concentrations of NO and TAC. Fifteen Ossimi rams, with an age range of 3–4 years, were selected from a group of 47 with the inclusion criteria being that A. that they were proven to be fertile (normal semen traits based on monthly semen analysis, and at least one confirmed pregnancy/ram during the last 6 months) and then B. that their BCS was in the various categories of 2–2.5 (lower BCS; *n* = 5), 3–3.5 (medium BCS; *n* = 5), and 4–4.5 (higher BCS; *n* = 5). The examined rams were selected in a completely randomised manner. The examined groups were blindly (ID identification only) subjected to testicular haemodynamics and echogenicity evaluation and blood retrieval for nitric oxide (NO) and total antioxidant capacity (TAC) measuring in the sera. The experimental procedures were carried out once per day on three separate days (i.e. 3 replicates). Each experimental day was separated by 2 days intervals.

#### Assessment of BCS

Rams’ body condition scoring was assessed following a previous study [11]. Briefly, in the loin area just before the last rib, the vertebral transverse and spinous processes were carefully palpated for the assessment of BCS. The scale used was from 1 (emaciated) to 5 (obese) and was measured in 0.5 increments.

### Assessment of testicular blood flow

**Fig. 1:**
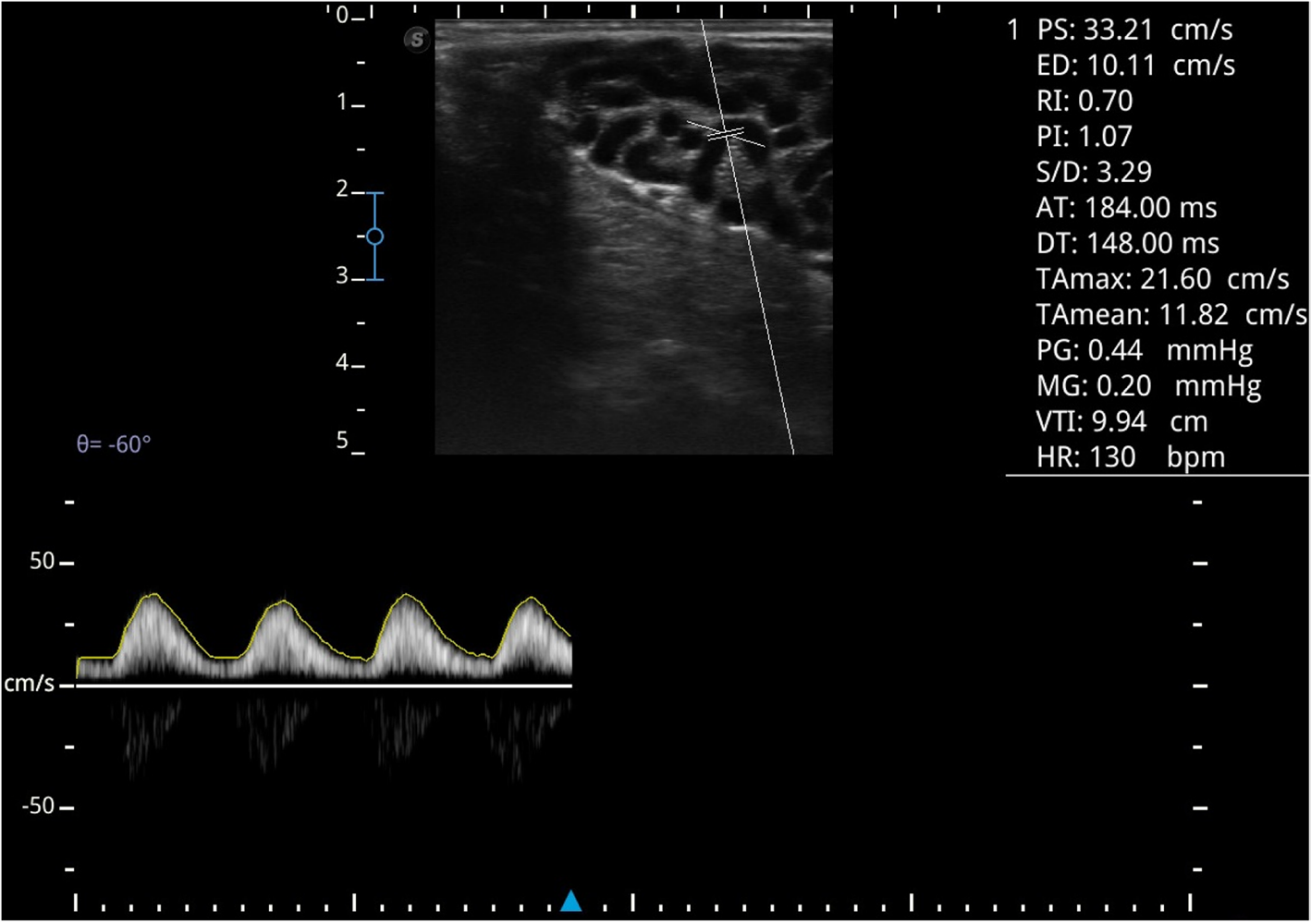
The spectral pattern of blood flow within the supra-testicular artery in Ossimi ram as assessed by Doppler ultrasonography indicates a monophasic and non-resistive waveform

Spectral Doppler ultrasonography of the supra-testicular artery (STA) was utilized to assess testicular perfusion (7–14 MHz, SonoScape E1V, SonoScape co., China). In detail, visualizing the testicular arteries of both testes demanded the removal of the scrotal wool and applying a suitable quantity of a sonographic gel followed by placing the device transducer (linear type) over the testicular attached end of the spermatic cord for visualizing the vascular cone of the STA. Upon detection of the STA vascular network, the Doppler gate was adjusted at 0.5 mm and directed to the STA lumen till the appearance of the spectral waveform (Fig. 1). After that, the image was frozen, and the Doppler parameters [peak systolic (PSV, cm/s), end-diastolic (EDV, cm/s), time-averaged maximum velocities (TAMAX, cm/s), pulsatility index (PI = PSV-EDV/mean velocity), and resistive index (RI = PSV-EDV/PSV)] were traced automatically by the Doppler device and recorded [18]. To avoid personal variations, all the ultrasound scanning was performed by one expert investigator with adjustment of the device settings (high-pass filter = 50 MHz; beam angle ≤60; brightness = medium) at the commencement of the study.

### Testicular echotexture assessment

**Fig. 2:**
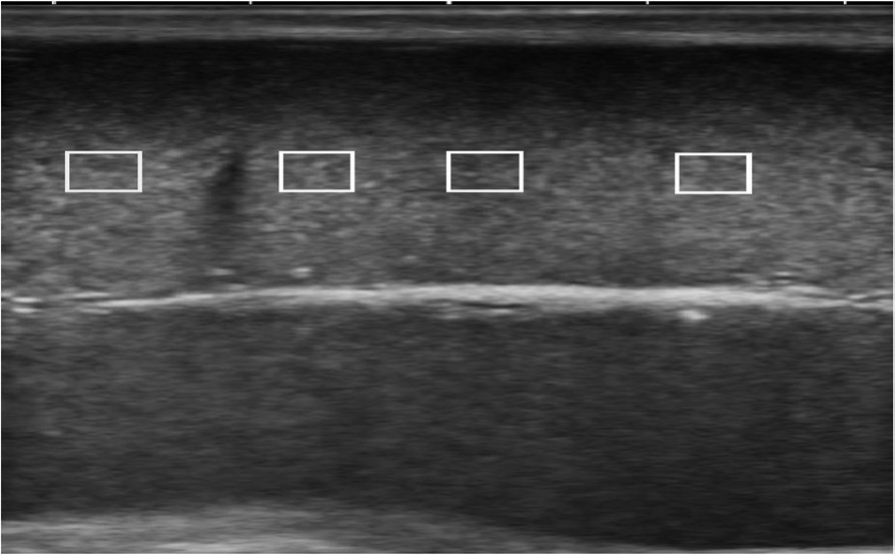
Ultrasonogram presenting an Ossimi ram testicular parenchyma, highlighting four squares (1*1 cm) over the highest echogenic line (mediastinum testes) for testicular echotexture measurement using Adobe Photoshop software

A B-mode ultrasound image of the testicular parenchyma was obtained by positioning the transducer on the longitudinal axis of the lateral surface of the examined testis until the mediastinum testis was in clear view. Testicular echotexture was evaluated using a software program (photoshop 64 cc, USA). In brief, at least three squares (1 cm * 1 cm/square) were created on the frozen images of the testis over the most hyperechogenic line (mediastinum testis; Fig. 2). The software calculated the pixel intensity (echotexture) and the standard deviation among these pixels (heterogeneity) of the selected area. The three squares’ average per testis was recorded and expressed as testicular echotexture and testicular heterogeneity [19].

### Serum harvesting and biochemical evaluations

Prior to each ultrasonographic assessment, blood sampling was performed by puncturing the jugular vein using a sharp (20 G) needle to fill a 5-ml plain collecting tube. A total of 3 blood samples were collected from each of the 15 rams on each day of examination, totaling 45 samples. Centrifugation of the collected blood was performed at 3000 rpm for at least 10 minutes followed by storage of the harvested sera in deep-frozen conditions (− 20 C^o^) until further assessments. After the study, the frozen sera were thawed and subjected to colorimetric evaluation of NO (in the form of nitrite) and TAC levels. Briefly, photometric kits (Batch: 210409 and 210,210; CAT.NO.: NO 2533 and TA 2513; Biodiagnostic Co., Giza, Egypt) were assigned using a spectrophotometer adjusted at 540 and 505 nm wavelength, respectively, with a sensitivity of 225 μM/L and 0.04 mM/L, and an intra-assay variation coefficient of 5.3 and 3.4%, respectively [20–22].

### Statistical analysis

To commence, the obtained data (3 replicates) were tested for normality (Shapiro-Wilk test) and homogeneity of variance (Levene test); *P > 0.05* for both tests was considered normally distributed and homogenous data. Testicular haemodynamic and echogenic data did not significantly differ between the right and left testis (*T*-test); therefore, it was pooled to be per/animal. The independent variable was BCS and the dependent variables were haemodynamic (PSV, EDV, RI, PI, and TAMAX) and echogenic (TE and PH) parameters, and serum concentrations of NO and TAC. Each dependent variable had 3 values per ram (as the left & right values were pooled) and each BCS cohort had 5 rams; therefore, the mean values of these 15 values (3 measurements per ram multiplied by 5 rams) were compared between each cohort group’s mean. One way ANOVA test was applied to show that there was a difference between the 3 groups and Tukey (post hoc) test was performed to show which group means differed using SPSS® 25, USA statistical software. Data were expressed as mean ± standard error of the mean (SEM) with *P < 0.05* considered significant.

## Results

### Association between BCS and testicular vascular dynamics and echogenicity

The data regarding the differences in the testicular vascular dynamics are depicted in Table 1. There was a significant (*P* < 0.05) difference in the RI and PI means among the groups under experimentation, being the least in the lower BCS rams (0.43 ± 0.03 and 0.57 ± 0.04, respectively) compared to the medium BCS (0.53 ± 0.03 and 0.77 ± 0.03, respectively) and higher BCS rams (0.57 ± 0.05 and 0.86 ± 0.03, respectively). Among blood flow velocities (PSV, EDV, and TAMAX), only EDV showed significantly (*P* < 0.05) higher values in the lower BCS group (17.06 ± 1.03 cm/s) compared to medium BCS (12.58 ± 0.67 cm/s) and higher BCS (12.51 ± 0.61 cm/s) groups. Regarding the TE results (Table 1), there were no significant differences among the examined groups.

**Table 1:** Mean values of the supra-testicular arteries’ Doppler indices, testicular parenchyma echogenicity, and sera concentrations of total antioxidant capacity (TAC) and nitric oxide (NO) in lower BCS (2–2.5), medium BCS (3–3.5) and higher BCS (4–4.5) rams

Parameters	Lower BCS group (n=5)	Medium BCS group (n=5)	Higher BCS group (n=5)
PSV (cm/s)	30.46 ± 1.99	29.21 ± 2.98	29.53 ± 1.23
EDV (cm/s)	17.06 ± 1.03^a^	12.58 ± 0.67^b^	12.51 ± 0.61^b^
TAMAX (cm/s)	22.71 ± 1.44	20.04 ± 1.69	19.77± 0.86
Resistive index	0.43 ± 0.03^a^	0.53± 0.03^b^	0.57± 0.05^b^
Pulsatility index	0.57 ± 0.04^a^	0.77 ±0.03^b^	0.86 ±0.03^b^
TE (pixels)	80.41 ± 4.82	74.51 ± 4.48	69.60 ± 5.09
PH	11.46 ± 0.39	10.33 ± 0.53	9.65 ± 0.61
TAC (mM/L)	0.90 ± 0.05 ^a^	0.58 ± 0.05^b^	0.45 ± 0.03^b^
NO (μM/L)	62.06 ± 2.72^a^	47.89 ± 1.49^b^	49.9 ± 3.63^b^

*PSV* peak systolic velocity, *EDV* end-diastolic velocity, *TAMAX* time average maximum velocity, *TE* testicular echotexture, *PH* pixel heterogeneity. Pulsatility index = PSV-EDV/mean velocity; resistive index = PSV-EDV/PSV. Data are presented as means ± SEM, with different superscripts ^(a-b)^ indicating significant differences at *P < 0.05*

### Association between BCS and concentrations of TAC and NO

The TAC (mM/L) and NO (μM/L) levels in different experimental groups are demonstrated in Table 1. There were significant differences (*P* < 0.01) in the concentrations of TAC and NO among the groups under experimentation, in which the lower BCS rams had the highest levels (*P* < 0.01) of TAC and NO in their sera (0.90 ± 0.05 and 62.06 ± 2.72, respectively) than the medium BCS (0.58 ± 0.05 and 47.89 ± 1.49, respectively) and higher BCS rams (0.45 ± 0.03 and 49.9 ± 3.63, respectively).

## Discussion

Evaluation of testicular blood flow provides useful insight into the haemodynamic patterns of testicular tissue. The present study illustrated an association between varying degrees of body condition and testicular haemodynamic parameters. Additionally, it also demonstrates that rams of various BCS categories were associated with varying systemic nitric oxide and total antioxidant capacity concentrations.

In the present study, the lower BCS cohort experienced the least values of RI and PI compared to the medium and higher BCS cohorts; this could be interpreted as decreasing the testicular artery resistance against the blood to flow toward the testicular parenchyma with subsequent higher blood perfusion [23, 24]. Literature exploring the association between BCS and reproductive performance in rams is scant. Assessment of BCS in the ram is nuanced and different from the ewe in terms of target BCS scores in different physiological conditions (mating, pregnancy, lambing, etc). It has been concluded that a BCS of 2.5–3 is the optimum in sheep production [25]. It has been reported that rams with BCS between 3 and 3.5 are optimum in terms of sexual behavior, semen quality, and testicular biometry under hot semi-arid conditions compared to BCS of 2.5 and 4 [11]. An earlier report indicated that a BCS of 4 is recommended for optimal reproductive performance in rams, and not less than 3 should be fixed all the year round [26]. Martin et al. [27] reported that a 150% increase in the nutritional plane (of the maintenance requirements) for a short period improved testicular volume and sperm output. However, it is not obvious that the improvement in semen quality is related to nutritional enhancement or BCS [28]. The discrepancy between the present study and the opposing study might be due to the differences in season (winter vs. summer), breed (Ossimi vs. Malpura), and target of exploration (testicular haemodynamics vs reproductive performance). Additionally, tail fatness seems to be another contributing factor; Ossimi rams have a fatty tail [29] compared to the thin-tailed Malpura breed [30]. Furthermore, thin-tailed Yankasa and fat-tailed Ossimi rams have quite different heat tolerances, with the latter being thought to be more sensitive. Therefore, fatty tail in Ossimi rams may be a source of mobilizable energy reserve that makes up for the reduced body condition in the lower BCS group. Explanation of the main cause of higher TBF in the lower BCS rams rather than the other groups is complicated; however, some of the evidence in the present study may partly explain this. The lower BCS rams had the highest NO and TAC levels among their counterparts. NO is primarily a vascular moderator, of endothelial origin and controls the vascular tone and function with a positive association with blood flow, i.e., higher NO levels induce vasodilatation and higher tissue perfusion [31, 32]. NO bioavailability is controlled by pleiotropic factors, among which the levels of free radicals (FR) are the main determinant. Higher levels of FR compulsively react with NO producing a highly oxidative molecule, called peroxynitrite, which induces oxidative stress concurrent with lower bioavailable NO and TBF [18, 33]. In addition, the lower TAC levels in the higher and medium BCS rams illustrate why the NO and TBF are lower than the lower BCS ones. Furthermore, it was reported that obese males experience a higher testicular temperature that increases their metabolic activity with subsequent higher ROS generation and lower TBF [15, 17]. In the present study, NO levels were assayed in the sera. As reviewed by Bryan and Grisham [34], blood assay of NO is not specific to definite tissue. It would be more beneficial to explore NO status in the testicular tissue; however and unfortunately, it was not accepted, by the farm policy, to apply a testicular biopsy.

The values of TAC, in the present study, were the highest in the lower BCS rams compared to other groups. It has been described that obesity triggers extra ROS generation and oxidative stress and debilitates the antioxidant defense systems [15]. The TAC results are corroborated by the NO levels being the highest in the lower BCS rams, as it decreased by the postulated overproduction of ROS in the fatty rams. TAC and NO levels, in the present study, support the haemodynamic patterns of the testicular arteries in the experimental groups that show concurrent higher TAC and NO levels with higher TBF.

Software-assisted testicular echogenicity (TE) is a useful and noninvasive tool for predicting semen quality in rams’ breeding soundness examination [35, 36]. TE scale ranged from 0 (black) to 255 (white) and varying grey colors in between, reflecting the cellular and fluid content of the testicular tissue. The present study reported a non-significant difference in echotexture of the testicular parenchyma among the rams with different BCS. It was reported that TE is correlated with the area of seminiferous tubules (ST) and their lumen; however, TE changes are not associated with variations in testicular haemodynamics [37–39]. In addition, it was reported a lack of relation between TE and semen traits in rams [40]. TE is mainly related to the cellular condensation within the ST rather than the numbers of ST and/or its lumen area or diameter [41]. The present study was performed during the breeding season when the rams are active and spermatogenesis is ongoing and this may be altering the echotexture.

The limitations of the present study were: (1) NO levels were assessed only in the serum samples instead of testicular tissue homogenate, (2) the sample size was relatively low; however, rams enrolled in the present study (15 out of 47) were only those which passed the inclusion criteria (BCS and fertility assurance); therefore, additional studies are required to be applied on a large ram population, (3) the study timeline was short, (4) we did not assess semen quality and fertilizing capacity.

## Conclusion

In conclusion, BCS was associated with TH and the antioxidant capacity of rams, with significantly lower indices of testicular dynamics found in rams in the lower BCS group as well as significantly higher levels of antioxidant capacity in this lower BCS group. Further research is required to evaluate the actual mechanism by which obesity affects TH on a molecular basis. It may be important to investigate the relationship between TH, semen quality, and fertility in rams with different BCS over various seasons, climates, and geographical locations.

## Data Availability

Data supporting the study outcomes are available upon request.
